# Diversity and evolution of anuran trypanosomes: insights from the study of European species

**DOI:** 10.1186/s13071-018-3023-1

**Published:** 2018-08-02

**Authors:** Viktoria V. Spodareva, Anastasiia Grybchuk-Ieremenko, Alexander Losev, Jan Votýpka, Julius Lukeš, Vyacheslav Yurchenko, Alexei Yu Kostygov

**Affiliations:** 1 Life Science Research Centre, Faculty of Science, University of Ostrava, Ostrava, Czechia; 2 Zoological Institute of the Russian Academy of Sciences, St. Petersburg, Russia; 3 Schmalhausen Institute of Zoology of National Academy of Sciences of Ukraine, Kyiv, Ukraine; 4 Department of Parasitology, Faculty of Sciences, Charles University, Prague, Czechia; 5 Biology Centre, Institute of Parasitology, Czech Academy of Sciences, České Budějovice, Czechia; 6 Faculty of Science, University of South Bohemia, České Budějovice, Czechia; 7 Martsinovsky Institute of Medical Parasitology, Tropical and Vector Borne Diseases, Sechenov University, Moscow, Russia

**Keywords:** Trypanosomatidae, Mixed infections, Evolution, Frog trypanosomes

## Abstract

**Background:**

Amphibian trypanosomes were the first ever described trypanosomatids. Nevertheless, their taxonomy remains entangled because of pleomorphism and high prevalence of mixed infections. Despite the fact that the first species in this group were described in Europe, virtually none of the trypanosomes from European anurans was analyzed using modern molecular methods.

**Methods:**

In this study, we explored the diversity and phylogeny of trypanosomes in true frogs from Europe using light microscopy and molecular methods.

**Results:**

A comparison of observed morphotypes with previous descriptions allowed us to reliably identify three *Trypanosoma* spp., whereas the remaining two strains were considered to represent novel taxa. In all cases, more than one morphotype per blood sample was observed, indicating mixed infections. One hundred and thirty obtained *18S* rRNA gene sequences were unambiguously subdivided into five groups, correspondent to the previously recognized or novel taxa of anuran trypanosomes.

**Conclusions:**

In this work we studied European frog trypanosomes. Even with a relatively moderate number of isolates, we were able to find not only three well-known species, but also two apparently new ones. We revealed that previous assignments of multiple isolates from distant geographical localities to one species based on superficial resemblance were unjustified. Our work also demonstrated a high prevalence of mixed trypanosome infections in frogs and proposed a plausible scenario of evolution of the genus *Trypanosoma*.

## Background

*Trypanosoma* is a genus of kinetoplastid flagellates enclosing more than 500 described species parasitizing all classes of vertebrates [1]. However, they were studied quite unevenly with the bulk of research focused on just two species causing severe diseases in humans, *T. cruzi* and *T. brucei* [2, 3]. To a much smaller (yet considerable) extent, attention has been paid to the economically important parasites of livestock (mostly *T. brucei evansi*, *T. brucei equiperdum*, *T. congolense* and *T. vivax*) as well as the non-pathogenic species *T. rangeli*, whose geographical distribution and host range overlap with those of *T. cruzi* [4–7]*.*

Other trypanosomes, i.e. those occurring only in wild animals, have been mostly neglected. Among them of a particular significance are amphibian parasites. This is the group, from which the study of the genus (and the whole family Trypanosomatidae) stemmed. Indeed, the first three species of trypanosomes were described from frogs: the type-species *Trypanosoma rotatorium* (Mayer, 1843) Laveran, 1901, as well as *T. loricatum* (Mayer, 1843) Dutton, Todd & Tobey, 1907, and *T. ranarum* (Lankester, 1871) Danilewsky, 1885. The early discovery of these trypanosomes was facilitated by their large size and, therefore, better visibility under the light microscope. Interestingly, this is the only group within the genus *Trypanosoma* demonstrating remarkable morphological plasticity. Besides classical fusiform trypomastigotes, there are rounded, oval, claviform, fan-shaped, leaf-like or irregular cells with or without a free flagellum, and longitudinal or spiral striations [8]. The morphology of the first three described species is so peculiar, that initially they were not even recognized as flagellates. *Trypanosoma rotatorium* was considered as an amoeba (*Amoeba rotatoria*), whereas *T. loricatum* and *T. ranarum* as ciliates (*Paramecium loricatum* and *Undulina ranarum*, respectively) [9, 10]. In other taxonomical groups, such a plethora of traits would simplify species delimitation and result in the well-established classification. However, pleomorphism (i.e. morphological changes during the life-cycle) and, apparently, mixed infections by different species of trypanosomes resulted in a taxonomic tangle. Many authors avoided describing new species and taking into account only simple superficial morphological resemblance applied some common names, ignoring the fact that their flagellates were distinct by a number of features and isolated from unrelated frog species and distant geographical locations [8, 11, 12]. In some cases, identifications were arbitrary and not substantiated by any analysis [13]. The name *T. rotatorium* was the most popular one, as flagellates from about 60 species of anurans from Europe, Asia, Africa, as well as North and South America have been recorded under this name [14–16]. All these reasons, along with rather limited efforts put in studying of amphibian trypanosomes, resulted in a relatively low number of species (about 60 in total) described by morphology. This number did not change significantly even after the advent of molecular phylogenetics.

Amphibian trypanosomes are very important from an evolutionary viewpoint. In accordance with the combined mode of life of their hosts, these parasites have both types of vectors: leeches as the trypanosomes of fish and dipterans as those of amniotes [17–19]. Therefore, this group was considered to be an intermediate, possibly even a connector between strictly aquatic (i.e. piscine) and strictly terrestrial (i.e. ungulate) trypanosomes [8]. Alternatively, having leeches as vectors was also regarded as an evidence of the independent origin of aquatic trypanosomes [20]. However, molecular phylogenetic reconstructions convincingly demonstrated monophyly of the genus *Trypanosoma* and its subdivision into two sister clades: aquatic and terrestrial trypanosomes [21–23]. In some reconstructions, the aquatic clade was split into the monophyletic “amphibian” and “fish + platypus + turtles” subgroups [17, 18, 24], while in other studies these relationships were unresolved [19, 25, 26]. In any case, the current state of knowledge prevents the unequivocal determination of the first vertebrate host of trypanosomes and reconstructing the directions of the subsequent radiation of these flagellates. However, a broader taxonomic sampling of aquatic trypanosomes could provide insights into these questions.

Despite the fact that the first species in this group were described in Europe, virtually none of the trypanosomes from European anurans were analyzed using molecular methods. The only exception was an isolate from the former Yugoslavia assigned (with no morphological evidence) to *T. neveulemairei* Brumpt, 1928 [27]. While several isolates from North America identified as *T. rotatorium* and *T. ranarum* were investigated by molecular methods [11, 28], it is unlikely that they are identical to the species described in Europe.

In this work, we explored the diversity and phylogeny of trypanosomes in true frogs from Europe, namely Ukraine and Czechia, using light microscopy and molecular methods.

## Methods

### Sample isolation and DNA extraction

Trypanosomes studied here were isolated from *Pelophylax ridibundus* and *P.* kl. *esculentus* collected in two locations in Ukraine and two locations in Czechia (Table 1). The presence of trypanosomes in frog blood was assayed on smears as described previously [29]. The smears containing trypanosomes were fixed with methanol, stained with Giemsa and used for subsequent investigation of morphology of the parasites. Three of the positive slides obtained from frogs from Bohemia, Czechia, also served as a source of DNA. For the other two Czech isolates, the available laboratory cultures were used for this purpose. As for the material from Ukraine, trypanosomes were isolated from the fresh blood of infected frogs by the microhaematocrite method [30] and used for DNA extraction as described elsewhere [31].

**Table 1:** Isolates of trypanosomes used in the present study. Parentheses denote the source of material for DNA extraction

Isolate	Blood sample	Smear	Culture	Geographical origin	GPS coordinates	Collection date	Host/prevalence of infection (if estimated)
R2	(+)	+		Oxbow lake of the Desna river, Vyshgorodsky district, Kyiv region, Ukraine,	50°36'49.8"N, 30°38'23.8"E	15/7/2014	*P. ridibundus*/8 out of 11 (73%)
R3	(+)	+				15/7/2014	
R4	(+)	+				15/7/2014	
R5	(+)	+				15/7/2014	
R6	(+)	+				15/7/2014	
R8	(+)	+				15/7/2014	
R10	(+)	+				9/10/2014	
R11	(+)	+				9/10/2014	
RrS1	(+)			Peat-bog near the village Rovzhi, Vyshgorodsky district, Kyiv region, Ukraine	50°56'09.0"N, 30°37'05.2"E	10/7/2015	*P. ridibundus*/3 out of 12 (25%)
RrS2	(+)					10/7/2015	
RrS3	(+)					10/7/2015	
Rer1		(+)		Natural reserve Ruda, South Bohemia, Czechia	49°09'2.61"N, 14°41'34.05"E	22/6/2003	*P*. kl. *esculentus*
Rer2		(+)				22/6/2003	
ZCZR1		+	(+)			23/6/2005	*P. ridibundus*
ZCZR2		+	(+)			27/6/2006	*P.* kl. *esculentus*
SKOKAN		(+)		Černičný pond, Lužnice, South Bohemia, Czechia	49°04'43.93"N, 14°45'11.04"E	21/6/2012	

### Morphological analysis

Since for the majority of samples blood smears were available, they were used to correlate the morphology of parasites with the obtained *18S* rRNA gene sequences. The smears were carefully inspected and every single trypanosome cell was photographically documented. The images were sorted according to morphotypes, counted and measured using Fiji software [32]. Several commonly accepted characters of frog trypanosomes were taken into account. Various numbers of cells were analyzed for particular morphotypes and sub-morphotypes depending on their availability and preservation quality. Not all of the considered features could be observed in some cells, mostly because of the high optical density of their cytoplasm.

### PCR, cloning and sequencing

The full *18S* rRNA gene was amplified from blood samples and cultures either as a single fragment using primers S762 and S763 [33], or in two overlapping fragments using the same two primers in combination with A757 and 883F, as described previously [29]. From DNA isolated from the blood smears, a ~900 bp long fragment of the *18S* rRNA gene was amplified with primers 1127F and 1958R [34]. All PCR products (except the homogenous ZCZ-R1 culture) were cloned and, typically, eight clones were sequenced for each isolate as described before [35]. The *gGAPDH* gene was amplified and sequenced for the monospecific culture ZCZ-R1 as described elsewhere [36]. The sequences obtained during this work were submitted to the GenBank database with the following accession numbers: MH424188-MH424313 (*18S*) and MH428670 (isolate ZCZR1 *gGAPDH*). *18S* rRNA gene sequences have originated from the following *Trypanosoma* sp. isolates: MH424188-MH424195 (R10); MH424196-MH424203 (R11); MH424204-MH424209 (R2); MH424210-MH424218 (R3); MH424219-MH424226 (R4); MH424227-MH424234 (R5); MH424235-MH424242 (R6); MH424243-MH424250 (R8); MH424251-MH424259 (Rer1); MH424260-MH424270 (Rer2); MH424271-MH424276, MH424291-MH424297 (RrS1); MH424277-MH424283 (RrS2); MH424284-MH424290 (RrS3); MH424298-MH424305 (SKOKAN); MH424306 (ZCZR1) and MH424306-MH424313 (ZCZR2).

### Phylogenetic analyses

The obtained *18S* rRNA gene haplotypes were aligned in MAFFT v.7 using the “Auto” algorithm [37] and the resulting alignment was manually inspected. One of the sequences contained an artificially duplicated 126 bp long fragment, which was manually deleted. The Bellerophon software [38] was used for chimera search both on the complete dataset as well as on the subset including only full-length sequences (in order to increase sensitivity).

To assess the distribution of haplotypes, a dendrogram was build using the GTR+G model and rapid hill-climbing algorithm in RAxML v.8.0 [39]. For each of the five observed groups a consensus sequence was inferred. These sequences along with those retrieved from GenBank for all frog trypanosomes and all main lineages of terrestrial and non-anuran aquatic trypanosomes were used for the phylogenetic tree inference. The sequences were aligned in MAFFT using the E-INS-i algorithm. The preliminary tree reconstruction demonstrated excessively long branches for some species (*Trypanosoma* sp. IAFR, *T*. *ranarum*, *T*. *chelodinae*, *T*. *neveulemairei*). Subsequent visual inspection of the alignment revealed that for these taxa some regions were misaligned likely due to multiple sequencing errors. Manual adjustments were made using the BioEdit v.7.2.5 program [40]. The alignment was then subjected to trimming using the “Automated1” algorithm in Trimal v.1.2 rev. 57 [41]. The final data matrix contained 100 taxa and 2127 sites. The maximum likelihood tree reconstruction was performed in IQ-TREE v.1.6 [42] with the best evolutionary model (TIM3e + I + G4) selected using Bayesian information criterion by the built-in ModelFinder [43]. Branch support was estimated using the standard bootstrap method (1000 replicates). Bayesian inference was accomplished in MrBayes v.3.2.6 under the GTR + I + G model, with analysis run for 5,000,000 generations, trees sampled every 1000 generations and other parameters left in default states [44].

The obtained *gGAPDH* gene sequence of the isolate ZCZ-R1 and those for other 12 trypanosome species (8 aquatic and 4 terrestrial) retrieved from GenBank were aligned with MAFFT. The resulting alignment was concatenated in Bioedit with that of the *18S* rRNA gene for the same taxa and used for phylogenetic inference in IQ-TREE and MrBayes. The analyses were done generally as described above, but with partitioning by gene and codon position for the *gGAPDH* gene. The best edge-linked partitioned model of nucleotide substitutions selected by ModelFinder was F81+F+I/TN+F+I/TPM3u+F/TIM3e+I+G4 for the first, second, and third codon positions of the *gGAPDH* and whole *18S* rRNA genes, respectively. This scheme was used in IQ-TREE, while in MrBayes it was relaxed to F81+I/GTR+I/GTR+I/GTR+I+G. All model parameters in Bayesian analysis were unlinked across all partitions, except branch lengths, which were linked by gene.

## Results

### Morphological analysis

Sixteen trypanosome isolates from *Pelophylax ridibundus* and *P*. kl. *esculentus* were analyzed (Table 1). Eleven of them were collected in the Kiev region of Ukraine: eight in an oxbow lake [prevalence of 73% (8/11)] and three in an acidic peat-bog [prevalence of 25% (3/12)]. Furthermore, five isolates from two localities in southern Bohemia, Czechia, were included (prevalence was not estimated). Large frog individuals (72–108 mm in length and weighing 53–126 g; corresponding to 3+ years of age) were selected for investigation.

**Fig. 1:**
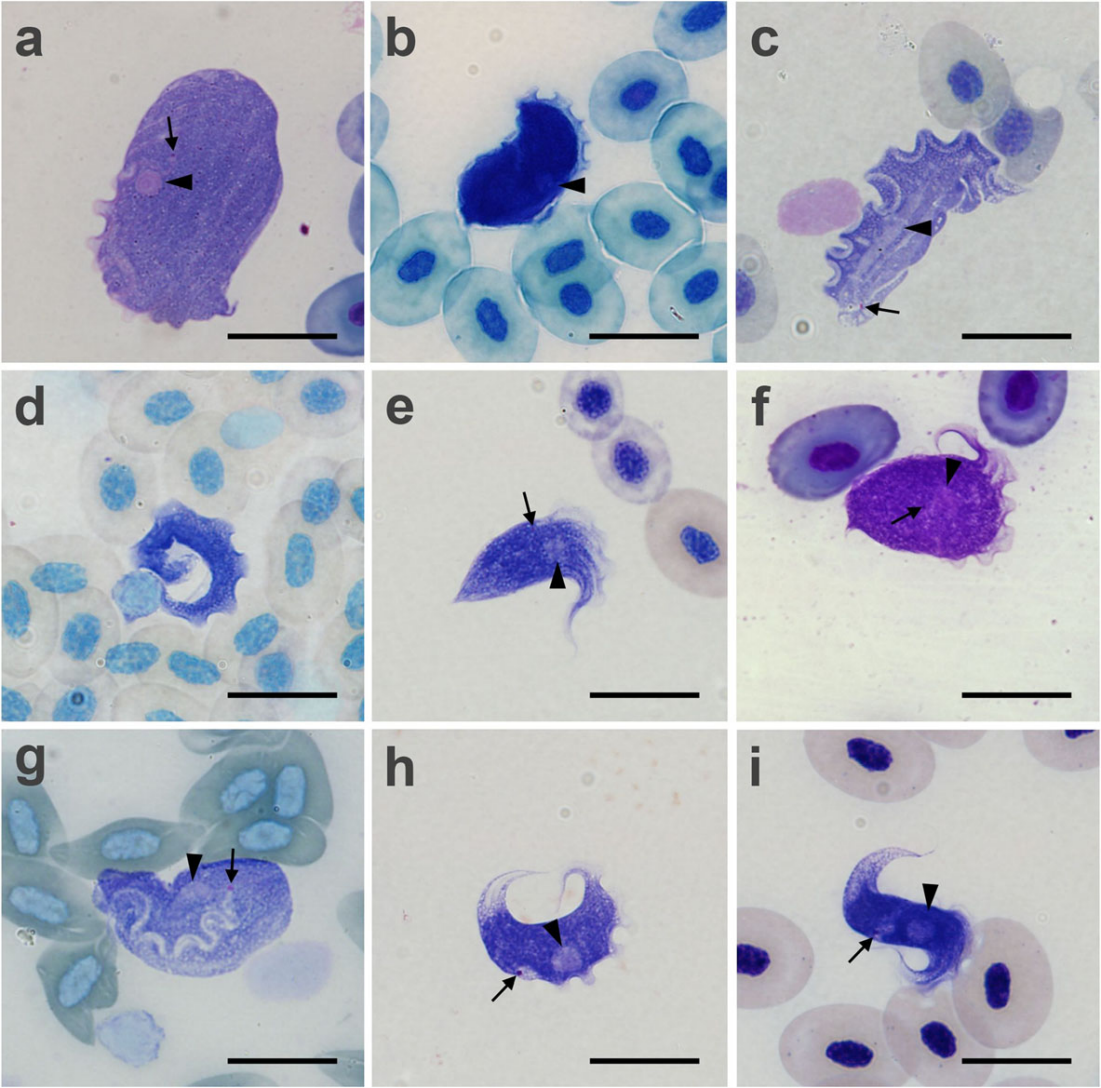
Trypanosomes observed in this work. **a** *Trypanosoma loricatum* (ReR2), normal form. **b** *T. loricatum* (ReR1), dense form. **c** *T. rotatorium* (R5), broad form. **d** *T. rotatorium* (R5), narrow (dense) form. **e** *T. ranarum* (ZCZ-R2), normal form. **f** *T. ranarum* (ReR2), broad form. **g** *Trypanosoma* sp. “nautilus”(R3). **h** *Trypanosoma* sp. “ring” (ReR1) crescent form. **i** *Trypanosoma* sp. “ring” (ZCZ-R2) S-shaped form. Arrows and arrowheads mark kinetoplasts and nuclei, respectively. *Scale-bars*: 20 μm

Trypanosomes encountered in the studied blood samples were morphologically diverse. In the Giemsa-stained smears we were able to distinguish five major morphotypes, as well as some variations thereof (Fig. 1). A comparison of these morphotypes with previous descriptions allowed a reliable species identification for three of them, whereas the remaining two strains were considered to represent putative novel taxa (Table 2). In all cases, we observed more than one morphotype per blood sample, indicating mixed infections. Below we describe the basic morphological features of the documented species and the differences from previous diagnoses.

**Table 2:** Morphometric characteristics of studied trypanosomes

	*T. loricatum* (*n* = 38)	*T. loricatum* dense form (*n* = 44)	*T. rotatorium* narrow form (*n* = 43)	*T. rotatorium* wide form (*n* = 20)	*T. ranarum* normal form (*n* = 45)	*T. ranarum* broad form (*n* = 6)	*Trypanosoma* sp. “nautilus” (*n* = 32)	*Trypanosoma* sp. “ring” (*n* = 26)
Cell length	35.7–76.0 (49.6 ± 7.1)	26.3–42.4 (36.1 ± 4.3)	39.0–63.0 (47.8 ± 5.3)	38.4–66.2 (50.0 ± 5.8)	36.8–52.7 (45.6 ± 3.3)	41.3–54.2 (47.2 ± 4.3)	29.3–61.6 (44.2 ± 7.7)	39.4–77.2 (61.7 ± 7.5)
Cell width	15.9–39.7 (25.4 ± 6.1)	12.0–24.6 (17.9 ± 2.9)	4.9–14.8 (7.9 ± 2.3)	9.6–27.6 (14.7 ± 4.0)	7.3–14.7 (11.4 ± 1.7)	19.1–22.7 (20.3 ± 1.4)	17.1–34.0 (23.2 ± 3.8)	5.8–12.6 (10.0 ± 1.7)
Nucleus length	4.3–5.9 (4.9 ± 0.4)	3.4–4.8 (4.1 ± 0.4) (*n* = 27)	–	11.6–23.7 (17.3 ± 4.1)	3.3–6.3 (4.5 ± 0.7) (*n* = 42)	5.0–7.4 (6.1 ± 0.9) (*n* = 5)	6.0–12.9 (9.9 ± 1.9)	4.3–6.6 (5.3 ± 0.6) (*n* = 26)
Nucleus width	–	–	–	1.9–4.5 (2.8 ± 0.6)	–	–	2.2–5.8 (3.8 ± 0.8)	–
Distance from anterior end to kinetoplast	22.9–47.2 (30.0 ± 4.5)	–	42.8–50.6 (45.6 ± 2.8) (*n* = 6)	39.6–58.1 (46.8 ± 4.1) (*n* = 16)	21.0–37.0 (29.9 ± 3.2) (*n* = 34)	33.1–44.1 (37.2 ± 4.9) (*n* = 4)	13.8–38.7 (26.7 ± 4.8)	16.5–38.3 (34.3 ± 4.7)
Distance from anterior end to nucleus	18.0–39.6 (24.0 ± 4.2)	14.8–22.3 (18.8 ± 2.1) (*n* = 26)	–	26.2–42.9 (33.8 ± 3.7)	18.5–33.6 (25.9 ± 3.2) (*n* = 42)	27.8–38.0 (30.5 ± 4.3) (*n* = 5)	9.0–30.1 (21.2 ± 4.1)	10.1–34.7 (27.5 ± 4.6) (*n* = 26)
Distance from nucleus to kinetoplast	4.6–8.6 (6.4 ± 0.9)	–	–	9.0–17.0 (13.4 ± 2.0) (*n* = 16)	3.1–7.7 (5.3 ± 1.0) (*n* = 34)	4.1–7.2 (6.4 ± 1.5) (*n* = 4)	4.0–8.9 (6.4 ± 1.3)	7.0–9.9 (8.4 ± 0.7) (*n* = 26)
Distance from posterior end to kinetoplast	9.3–28.8 (19.6 ± 4.5)	–	1.2–3.0 (2.3 ± 0.7) (*n* = 6)	1.7–4.9 (2.9 ± 0.9) (*n* = 10)	7.0–20.3 (15.4 ± 3.2) (*n* = 34)	10.1–14.2 (12.1 ± 2.0) (*n* = 4)	10.7–27.7 (17.2 ± 4.7)	18.7–39.0 (27.5 ± 4.8) (*n* = 26)
Height of undulating membrane	1.2–7.5 (3.1 ± 0.9)	1.6–3.9 (2.8 ± 0.5) (*n* = 35)	1.2–2.2 (1.7 ± 0.3) (*n* = 14)	0.9–1.8 (1.4 ± 0.3) (*n* = 18)	3.0–5.5 (4.2 ± 0.6) (*n* = 45)	3.8–4.8 (4.2 ± 0.5) (*n* = 6)	–	1.5–5.0 (3.0 ± 0.7)
No. of undulations	–	–	5.0–11.0 (6.6 ± 1.3) (*n* = 24)	5.0–11.0 (7.1 ± 1.5) (*n* = 16)	–	–	–	–
Free flagellum length	–	–	31.0–58.3 (38.8 ± 8.6) (*n* = 8)	32.9–62.6 (46.6 ± 14.2) (*n* = 4)	6.3–21.1 (11.5 ± 3.5) (*n* = 40)	6.5–15.0 (9.7 ± 3.1) (*n* = 6)	8.2–11.7 (10.0 ± 1.5) (*n* = 5)	10.5–26.0 (19.7 ± 4.4) (*n* = 10)

*Notes*: All measurements are in micrometers. The number of measured cells is indicated in the heading, but particular characters could be observed only in a portion of individuals (indicated in the corresponding table cells)

*Trypanosoma loricatum* was represented by oval or slightly elongated large cells with longitudinal or oblique striation. The round nucleus was situated laterally in the central part of the cell. The small kinetoplast was located close to the nucleus and was often hardly discernible. The free part of the flagellum was short and, in many cases, not visible (Table 2). The height of the undulating membrane did not exceed the diameter of the nucleus. This species displayed three previously characterized forms [45]: normal (Fig. 1a); elongated (not shown, differing from the former only in cell shape); and dense (Fig. 1b). The latter variant featured a dark staining of the cytoplasm, which prevented observing kinetoplast, nucleus and striation. This species was originally described from *Pelophylax* kl. *esculentus* in Germany [9].

*Trypanosoma rotatorium* had a leaf-like cell shape with well-developed undulating membrane. The long sausage-like nucleus was usually well discernible, shifted to the posterior end of the cell and located close to the kinetoplast. The latter was in subterminal position and, despite its small size, was usually conspicuous. In contrast to other species, the undulating membrane plicae in this trypanosome were of the same color as the cytoplasm. The free flagellum was hardly visible, representing 69–131% of the body length (89% on average). However, within the undulating membrane the flagellum was prominent as a wavy light line (Table 2). This species was represented by a transparent broad form (Fig. 1c) and a dense narrow form (Fig. 1d). The latter possessed a darkly stained cytoplasm with an indiscernible nucleus and kinetoplast as well as a shorter free flagellum. The original description of this species was performed using the blood samples of *Pelophylax* kl. *esculentus* from Germany [9]. Canadian isolates from *Lithobates clamitans*, *L. catesbiana* and *L. pipiens* assigned to the same species [46, 47] can be distinguished by the following characters: (i) round nucleus; (ii) kinetoplast situated in about one-fifth of the body length from the posterior end; and (iii) free flagellum absent. The *18S* rRNA gene sequences for two of these isolates from *R. catesbiana* were previously deposited in the GenBank database [22, 33].

*Trypanosoma ranarum* displayed conical cells with a fan-shaped anterior portion and a pointed posterior end. The widened anterior part bore prominent longitudinal ridges and was bordered by the plicae of the undulating membrane. The latter ended with a tapered rostrum situated laterally on cell’s anterior end. The maximal width of the plicae of the undulating membrane was comparable with the diameter of the nucleus, which was round and situated in the anterior part of the cell. The kinetoplast was small and located close to the nucleus. The free flagellum was short, 13–49% (25% on average) of the cell length (Table 2). This species was represented by a normal (Fig. 1e) and a broad form (Fig. 1f). The latter was characterized by a wide cell body and a short free flagellum. In some broad cells, the posterior end was blunt. This species was originally described from *Pelophylax* kl. *esculentus* in Germany [10]. The Canadian isolate assigned to the same species [47], is different in that it has a crescent shape, a long tapered posterior end, and a kinetoplast situated in the posterior half of the cell. This isolate had been deposited to the ATCC and its *18S* rRNA gene sequence is available from GenBank [27].

*Trypanosoma* sp. “nautilus” had a variable body shape (other authors described it as oval or rounded [15, 45]), but often it was reminiscent of the cephalopod mollusk *Nautilus pompilius*, wherefrom originates our tentative name for this flagellate. The cell was typically pointed on the anterior end to form a claw-like appendage where the free flagellum emerges. The nucleus was usually oval, occasionally drop-like or rounded. The kinetoplast was situated close to the nucleus, sometimes almost adjacent to it. The free flagellum was short (22–30%), 25.5% on average of the body length (Fig. 1g, Table 2). A very similar flagellate, *T. nagasakiense*, had been described in Japan from *Hyla arborea japonica* [48]. However, the trypanosome described here is larger than *T. nagasakiense*: body length 29.3–61.6 (44.2 ± 7.7) μm *versus* 24.0–39.0 (33.0) μm; body width is 17.1–34.0 (23.2 ± 3.8) μm *versus* 15.6–20.4 (18.1) μm. Considering the unrelated hosts (belonging to distinct families) and different geographical origin of *Trypanosoma* sp. “nautilus” and *T. nagasakiense*, we consider that they represent separate species. However, the absence of molecular sequences for *T. nagasakiense* does not allow for confirmation of this suggestion. A similar trypanosome was recorded in Brazilian anurans of the families Hylidae and Leptodactylidae [15]. Although in that work a molecular phylogenetic analysis was performed, the *18S* rRNA gene sequences were not associated with the observed morphotypes.

*Trypanosoma* sp. “ring” had a fusiform body tapered and pointed at both ends. The posterior end was extremely narrow and formed a hair-like structure. In the posterior portion of the cell, the cytoplasm was transparent and bore a prominent longitudinal striation. In some narrower individuals, this striation could also be observed in the anterior part of the cell. The nucleus was large, round and located in the anterior half of the cell. The kinetoplast was situated laterally in the central part of the cytoplasm. In the front of the kinetoplast or, less frequently, around it there was a conspicuous light oval zone. The flagellum was short (15–57%, 32% on average) of the body length. The cells appeared to be flexible. In most cases, they had a crescent profile and some of the narrower ones even formed an almost closed circle (Fig. 1h). Less frequently, cells bent in opposite directions (and thereby producing an S-shaped form) were observed (Fig. 1i). Supposedly, the same species had been documented as “*T. rotatorium* form 4” in *Rana temporaria* from Lithuania [49] and “*Trypanosoma* sp. 3” in *Pelophylax ridibundus* and *Rana amurensis* in Kyrgyzstan [45]. A trypanosome very similar in measurements and overall body shape tentatively identified as *T. bufophlebotomi* had been documented in *Bufo americanus* from the USA [50]. Nevertheless, this parasite differs from *Trypanosoma* sp. “ring” in the close proximity of its nucleus and kinetoplast, as well as in the absence of the broad light zone in the posterior part of the cell. Given that the spindle shape is very common among trypanosomes, many previously described species superficially resemble *Trypanosoma* sp. “ring” characterized here.

### Phylogenetic analyses

**Fig. 2:**
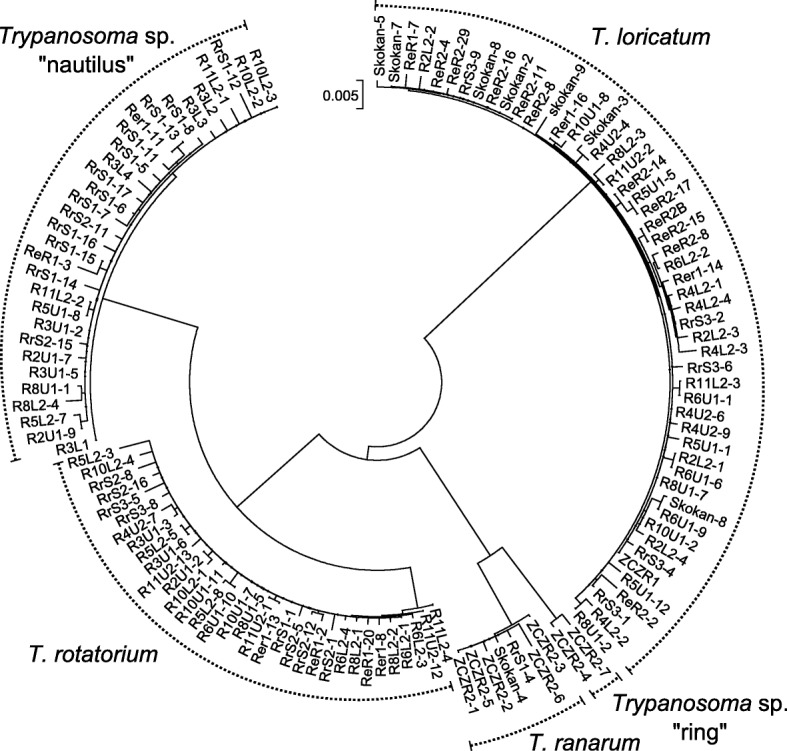
Dendrogram of the obtained *18S* rRNA gene sequences. The numbers following dashes in the sequence names refer to molecular clones. The scale-bar indicates the number of substitutions per site

Sequences were obtained from cloned PCR products from DNA from cultures or slides. From the 130 obtained *18S* rRNA gene sequences, four were identified as chimeras, while the remaining sequences could be unambiguously subdivided into five groups (Fig. 2). In most cases, the differences among the sequences within a group did not exceed 0.005 substitutions per site, which could be attributed to the combination of intraspecific variability and PCR errors. Given that the number of haplotype groups coincided with that of the observed morphotypes, we correlated them based on the presence/absence and relative abundance within each particular sample. This allowed us to assign sequences to a particular species. The most frequent haplotypes were those of *T. loricatum* (41%), followed by *T. rotatorium* (27%) and *Trypanosoma* sp. “nautilus” (25%), whereas *Trypanosoma ranarum* and *Trypanosoma* sp. “ring” were rare (6.0% and 1.5%, respectively). The sequences for the latter trypanosome were present only in one specimen from Czechia, while the four other species were detected in both Czech and Ukrainian samples. Virtually all samples displayed mixed trypanosome infections. The only exception was the culture ZCZ-R1, which contained only *T. loricatum*.

**Fig. 3:**
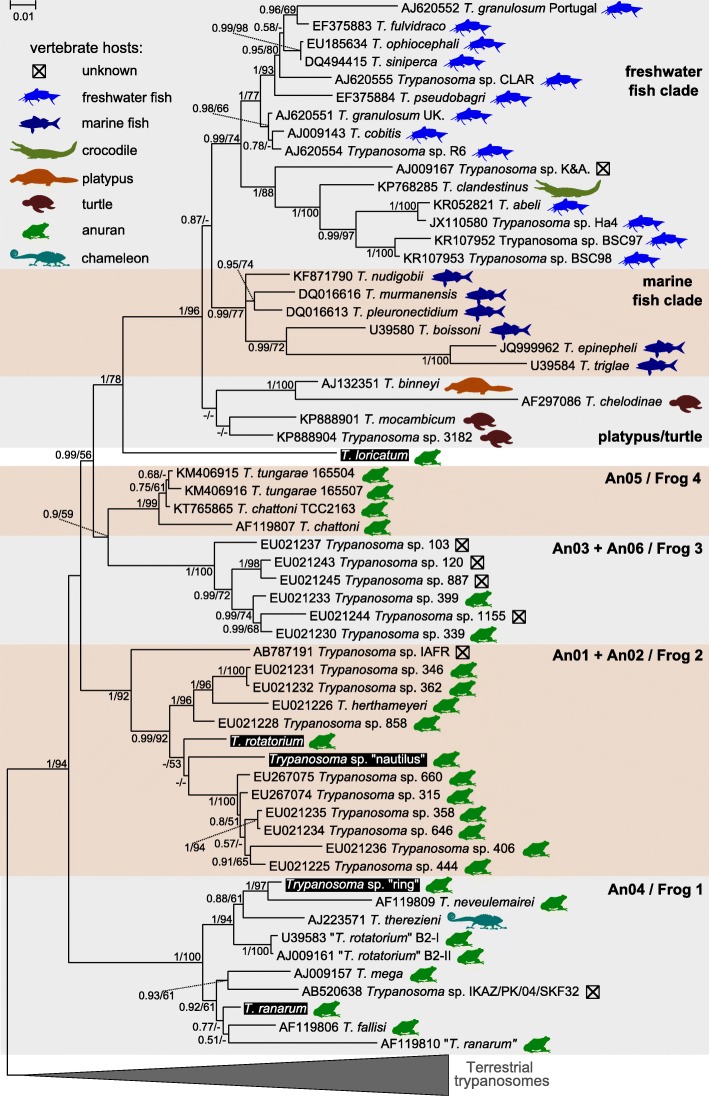
Maximum likelihood phylogenetic tree of aquatic trypanosomes based on *18S* rRNA gene sequences. Host taxa are shown by symbols defined in the key for hosts. The clades of frog trypanosomes are labeled according to [19, 51]. The species studied in the present study are marked by inverted colors of font and background. Numbers at the branches indicate the Bayesian posterior probability and maximum likelihood bootstrap support, respectively. The scale-bar indicates the number of substitutions per site. The tree was rooted with the sequences of terrestrial trypanosomes. The accession numbers for the studied trypanosomes are not indicated since in each case the consensus inferred from several sequences was used

The consensus sequences for each of the five species were used in phylogenetic inferences. In general, the reconstructed tree was congruent with those published earlier and we were able to delineate the same groups Frog 1-Frog 4 [19]. However, we observed some discrepancy with the alternative classification of the clades (An01-An06) proposed by Brazilian colleagues [51]. In our inference (as in [19]) the groups An03 and An06 could not be separated from each other, since the species were intermingled (Fig. 3).

*Trypanosoma* sp. “ring” and *T. ranarum* were placed in the group Frog 1 (= An04), which contained several species described previously. Specifically, the *T. ranarum* described here was closely related (but not identical) to an isolate from the USA identified as *T. ranarum* and *T. fallisi* from Canada. However, the insufficient tree resolution (and apparently low quality of the previously reported sequences) did not allow us to assess these relationships with certainty. *Trypanosoma* sp. “ring” formed a well-supported clade with *T. neveulemairei* from the former Yugoslavia.

Two other species, *T. rotatorium* and *Trypanosoma* sp. “nautilus”, were nested within the clade Frog 2 (or An01 + An02) formed by many undescribed species of anuran trypanosomes from Brazil, isolate IAFR from Ghana, as well as *T. herthameyeri* from Brazil. Although both species appeared to be more related to the An01 group, the corresponding statistical support was moderate. Importantly, the European isolate of *T. rotatorium* described here was distant from the two Canadian isolates designated as *T. rotatorium*, which resided in the Frog 1/An04 clade.

**Fig. 4:**
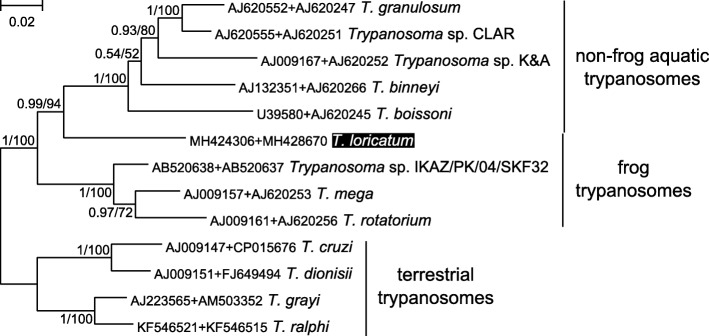
Maximum likelihood phylogenetic tree of trypanosomes based on concatenated *18S* rRNA + *gGAPDH* gene dataset. The species studied in the present work is marked by inverted colors of font and background. Numbers at the branches indicate the Bayesian posterior probability and maximum likelihood bootstrap support, respectively. The scale-bar indicates the number of substitutions per site. The tree was rooted with the sequences of terrestrial trypanosomes

*Trypanosoma loricatum* formed a sister clade to the fish/turtle/platypus/crocodile trypanosomes, thereby rendering the frog trypanosomes paraphyletic. While the posterior probability of this relationship was maximal, the bootstrap support was only moderate. Therefore, we tested this hypothesis using a concatenated *18S* rRNA and *gGAPDH* gene dataset, which was significantly smaller given the low number of the available *gGAPDH* gene sequences for the aquatic trypanosomes. The combined dataset supported the same topology with a high bootstrap value (Fig. 4).

## Discussion

European frog trypanosomes are a scarcely investigated group, which has not been assessed by molecular methods thus far. In this study, even with a relatively moderate number of isolates, we were able to record not only three described species, but also two apparently new ones. *Trypanosoma* sp. “nautilus” has not been previously recorded in Europe, but is morphologically similar to the Japanese species *T. nagasakiensis*. However, the very long distance between the localities of isolation, differences in host specificity and discrepancies in size suggest that these two trypanosomes are distinct species. *Trypanosoma* sp. “ring” could not be assigned to any described species at all. This demonstrates that even in Europe, with its modest number of anuran species, the limits of known diversity of frog trypanosomes are far from being reached.

Our study also demonstrated a high prevalence of mixed trypanosome infections in frogs; specifically, each of the studied blood samples contained more than one species. Although co-infections were already reported from various aquatic vertebrates including anurans [12, 15, 24, 26, 31, 52], to the best of our knowledge, this is the only published dataset with such a high prevalence of co-infections. Interestingly, this concerned even the frogs from an acidic peat-bog, where the overall infection rate was rather limited (only 25%). We speculate that different trypanosome species may have the same vector and, thus, be transmitted simultaneously. The phenomenon of mixed infections might have misled many researchers in the past, who considered various simultaneously encountered species as one, thereby exaggerating the extent of pleomorphism [49, 53, 54].

Another important conclusion concerns the morphology of anuran trypanosomes. As already mentioned above, this group of parasitic protists is characterized by a wide range of forms, theoretically enabling easy discrimination of species. However, using a broad interpretation of diagnostic traits, some researchers assigned unrelated isolates to a single species. Here, we demonstrated that the genuine *T. rotatorium* from Europe is not only morphologically, but also phylogenetically distinct from the two known isolates from Canada. Because *T. rotatorium* was originally described from Europe, it is clear that the Canadian isolates represent distinct, so far undescribed, anuran trypanosomes.

A very similar situation occurs also in the case of American “*T. ranarum*”. It also differs from the European counterparts, although these turned out to be closely related, as judged by molecular data. We believe that in anuran trypanosomes, morphology is of limited value for assessing species relatedness. The most striking example is the phylogenetic proximity of *T. chattoni* and *T. tungarae*, displaying round cells with rudimentary flagellum and typical serpentine trypomastigotes, respectively [19]. It is worth mentioning that even in this case there may be a confusion, since *T. chattoni* was originally described in Vietnam, whereas the *18S* rRNA sequences are available only for an ATCC-deposited culture originating from the USA [27] and an isolate from Brazil [25].

*Trypanosoma loricatum* remained neglected for a long time. Given its simultaneous description with *T. rotatorium*, many authors considered it just another developmental stage of that species [54]. Furthermore, modern studies of anuran trypanosomes were carried out mainly by researchers from the New World, where this and similar species are apparently absent. Meanwhile, *T. loricatum* proved to be very important from the evolutionary viewpoint. Here, we demonstrated that this species represents a sister group to the clade of non-frog aquatic trypanosomes, thereby making anuran parasites paraphyletic, i.e. being ancestral to them. Apparently, such a transition may have occurred owing to a common vector of both groups, the leeches. Most anuran trypanosomes are transmitted by dipterans, but some of them utilize leeches as vectors [8, 55]. In the current work, we did not establish the invertebrate hosts for the studied trypanosomes. However, it was reported that for *T. costatum* (synonym of *T. loricatum*) leeches may serve as vectors [56, 57]. Thus, the origin of non-frog trypanosomes seems to be straightforward: an ancestral leech-transmitted anuran parasite has adapted to new host groups (fish and aquatic amniotes). However, speculations on this subject give rise to several important questions. Firstly, in the current phylogenetic reconstruction this transition appears unique, although *T. loricatum* is not the only leech-transmitted anuran trypanosome. Why in other lineages such host switching did not occur? Secondly, why could anurans be the original trypanosome hosts? Thirdly, how are the terrestrial trypanosomes related to the aquatic ones? To answer all these questions, one should consider the evolution of the whole family Trypanosomatidae. The discovery of the mosquito-dwelling flagellate *Paratrypanosoma confusum*, which represents the earliest branch within the family [58, 59], provided evidence that first trypanosomatids were monoxenous parasites of insects, most likely dipterans. The switch to the dixeny was enabled by their invasion of blood-sucking hosts [60, 61]. We propose that Amphibia became primary vertebrate hosts, since during metamorphosis their immune system undergoes dramatic reorganization, rendering them more susceptible to infections [62]. This was not an easy transition, as judged by its presumable singularity. The subsequent radiation within anurans apparently prepared the ground for the expansion of trypanosomes to other vertebrate groups. However, due to substantial immunological differences of the hosts, such transitions were rare. We can now assert that those were the above-discussed switches to fish, platypuses, turtles and a unique case of *T. therezieni*, a parasite of the chameleon *Calumma brevicorne* (Fig. 3) [63]*.* The proposed evolutionary scheme suggests that the terrestrial trypanosomes also originated from the amphibian parasites. However, the currently available data do not allow thorough testing of this hypothesis. A wider sampling of the amphibian trypanosomes and/or the inference of multigene phylogenies may prove their paraphyletic status also in relation to the terrestrial clade.

## Conclusions

The application of morphological and molecular methods to the study of European frog trypanosomes revealed relatively high species diversity in spite of the moderate number of isolates: three described species and two putative new species. In addition, we demonstrated that trypanosomes isolated in distant geographical localities and having superficial resemblance had been erroneously assigned to the same species. Our study also demonstrated a high prevalence of mixed trypanosome infections in frogs and proposed a plausible scenario of evolution of the genus *Trypanosoma*. We propose that an ancestral leech-transmitted anuran parasite has adapted to the new host groups (fish and aquatic amniotes) giving rise to all other trypanosomes.
